# What is better for psychiatry: Titrated or fixed concentrations of nitrous oxide?

**DOI:** 10.3389/fpsyt.2022.773190

**Published:** 2022-08-22

**Authors:** Mark A. Gillman

**Affiliations:** Directorate, South African Brain Research Institute, Johannesburg, South Africa

**Keywords:** depression, psychiatry, titrated nitrous oxide, fixed concentrations nitrous oxide, ketamine, substance abuse, alcohol withdrawal

## Abstract

Medication dosages are crucial–no single dose fits all. My paper compares the safety, scientific and practical applicability of fixed 25–50% concentrations of nitrous oxide (N_2_O) with the variable titrated concentrations of Psychotropic Analgesic N_2_O (PAN), as used in dentistry, and neuropsychiatry. A crucial difference is that PAN is always titrated, *via* an open circuit (nasal mask), to the *minimum* concentration (dose), which ensures full consciousness, cooperation, comfort and relaxation. With PAN, the goal is subject comfort, not dose. In contrast, fixed goal concentrations are usually given *via* relatively closed circuits (full facial mask/similar) without account for individual patient's dose-response. Hence, fixed concentrations, in N_2_O sensitive subjects, could result in unconsciousness and other adverse effects (nausea, vomiting, anxiety, aspiration, might occur; requiring an anaesthesiologist for patient safety. PAN is titrated using each subject's subjective and objective responses as the guide to the ideal concentration. Thus, when PAN is used, there is no fixed concentration even for a single subject, nor is an anaesthesiologist required. Furthermore, there is a greater scientific rationale for using PAN, because the receptor systems involved are better known, whilst those for fixed concentrations are not. The PAN or dental titration method has been safely used in general dentistry for over 70 years and as an investigative, diagnostic and therapeutic tool for neuropsychiatry for over 40 years. Clinical applications include substance abuse detoxification, ameliorating depression, and investigations of schizophrenia, human orgasm, pain perception and basic neuroscience. By contrast, the experience with fixed doses in psychiatry is limited.

## Introduction

There is a sudden reawakening of interest in using subanaesthetic concentrations of N_2_O in psychiatry (1–6). Thus, we need answers to these questions. Particularly, as research has already shown the greater safety (7, 8) and wider usefulness of correctly titrated subanaesthetic N_2_O for psychiatry (9–12).

The technique recently advocated (1, 2, 4, 6), a fixed 50% concentration of N_2_O (5). Unfortunately, this is less safe (7, 8, 13), unless used in hospital practice, with an attending anaesthesiologist. Importantly, the only past experience with 50% N_2_O in psychiatry is anecdotal and limited to a few patients only (14).

In a later double-blind study, Nagele et al., using 50% found that 25% N_2_O was safer and yet as effective and reduced the unwanted side effects considerably (15). However, like 50%, 25% is not tailored to each subject's needs. As a result, those sensitive to the actions of N_2_O, will be unnecessarily exposed to side-effects (7, 8) including the undesirable psychomimetic states, found with ketamine (6, 16). Inadvertently, by using different dosages, these authors have underlined the dose-dependent variability of the effects of N_2_O.

The other technique, PAN (psychotropic analgesic nitrous oxide) refers to *low* subanaesthetic concentrations of N_2_O as used in modern dentistry (7, 8, 17). Inhalation sedation or minimal sedation refer to the identical dental technique as PAN (7, 8, 11, 17, 18). PAN concentrations are titrated to the point of the subjects' maximum comfort and effectiveness (and are not fixed at any specific level) and therefore vary, depending on the each subject's sensitivity to the gas.

The main objection to using fixed concentrations, whether 25% or 50% is that the majority of individuals inhaling N_2_O will be subjected unnecessarily (7) to unwanted side-effects such as nausea, vomiting, sleepiness and headache (6–8, 15, 16). Indeed, in hypersensitive people sleepiness could be converted to anesthesia (7, 8) as well as other undesirable psychomimetic effects, including delusions, anhedonia, mania and paranoia as well as distortions in perception, mental ineffectiveness and anhedonia (6, 15, 16). Likewise, at both the fixed 25% or 50% levels, many patients will not have received the optimal dosage. In both the latter scenarios, giving too high or low a dose is undesirable. In addition, only a fraction of patients are likely to receive the correct concentration.

This paper will briefly discuss some of the research conducted with PAN and fixed concentrations and then compare their advantages and disadvantages (see Table 1).

**Table 1:** Comparison of fixed and variable titrated (PAN) concentrations of subanaesthetic N_2_O for psychiatry.

	**Subanesthetic fixed N_2_O concentrations**	**Titrated N_2_O (PAN) concentrations variable**
Relation to Guedal's stage 1 anesthesia	Upper half Stage 1 (19) Disadvantage: Fixed concentrations; relatively high (relatively closed circuit with facial mask) (1–5, 15, 19).	Lower half Stage 1 (19) Advantage: PAN concentration always lower (relatively open circuit) (17, 19–21).
Scientific rationale and receptor mechanism	Convenience and standardization. Psychiatric use in single, past anecdotal study (14). Not used in dentistry (7, 8, 12, 13, 17) or usually by continuous flow in obstetrics (22), despite contrary statement (1, 23). Receptor mechanism uncertain, depends on subject's N_2_O sensitivity (7, 8, 12, 15, 17, 24–28). Advantages: Fixed concentration; 1. Convenient and 2. Standardized. Disadvantages: Fixed concentrations; <35% cause 1. Anesthesia in ≈30% of subjects (24–26) and 2. Receptor involved uncertain; NMDAR (29, 30) or >35 opioid & GABA-ergic systems (12, 27, 29, 31–33).	Based on usefulness and safety in psychiatry for 40 years (8–12, 34–36) and in dentistry for even longer (7, 8, 11, 12, 17, 37). Receptor mechanism more certain (29, 31–33, 38, 39) because subjects' N_2_O sensitivity always considered and subjects always conscious (7, 8, 12, 13, 17, 29, 36). Advantage: PAN: Receptor mechanism certain; opioid and GABA-ergic systems (11, 12, 28, 29, 31–33, 36, 38, 39). Disadvantages: PAN; 1. Concentration varies and 2. Needs; A. Careful titration and B. Special training (7, 8, 17, 36, 37, 40, 41).
Anesthesiologist + Standard ASA monitoring equipment.	Anesthesiologist required (1, 4, 5, 15). Disadvantages: Fixed concentrations; 1.Costly because A. Two practitioners required (anesthesiologist and psychiatrist) plus B. Expensive monitoring equipment (1, 4, 5, 18).	Anesthesiologist not required (7, 8, 17, 18, 37). Advantages: PAN; Less costly; 1. Single-handed practitioners able to administer the gases (provided they are trained). A. No anesthesiologist plus B. Additional monitoring equipment not needed (6, 7, 17, 36, 37).
Circuit (Crucial)	Semi-closed–full face mask, strapped to face covering nose and mouth^†^ (1–5, 15). Disadvantages: Fixed concentrations; Semi-closed circuit –discomfort and anxiety common in normal; probably worse in psychiatrically compromised (1, 7, 8, 13, 15, 27, 42).	Semi-open–nasal mask(hood); passively (loosely) applied (no straps)– mouth has access to room air^†^ (7, 8, 17, 27). Advantages: PAN; Semi open circuit– adjusted to patient comfort and anxiolytic (7, 8, 10–13, 17, 27).
Titration	To goal concentration (1–3, 5, 15). Disadvantage: Fixed concentration; side effects unavoidable in some (7, 8, 13, 15, 24, 26, 27, 42).	To subjects level of comfort (7, 8, 10, 11, 17). Advantages: PAN; Side effects usually avoided rarer and milder (6, 7, 11, 13, 27).
Goal	Constant concentration (1–5, 15). Disadvantages: Fixed concentration; Some subjects uncomfortable because comfort secondary to goal concentration (1–5).	Never goal–concentrations, always variable and titrated to patient comfort (6, 7, 9–11, 17, 36). Advantages: PAN; subject always comfortable (6, 7, 10, 11, 17).
Concentration achieved in pharynx	Always >35% N_2_O (semi- closed circuit). Disadvantages: Fixed concentration: Side-effects in some and more severe (1, 5, 7, 8, 13, 15, 24–27).	Always >35% (20, 21). Advantages: PAN: Side-effects usually avoidable and when occur rarer and milder (7, 8, 24–27).
Safety and recovery	Only safe if given by anesthethesiologist (1, 4, 15, 18, 38). Disadvantages: 1.safe to drive only after >30 mins^††^ because of higher concentration and longer exposure (13, 19, 24, 25, 43). 2. Anesthesiologist must be present, to ensure safety (1, 4, 5, 18) because ≈30% of subjects likely to be anesthetized (24–26).	Safe without anesthesiologist present (7, 8, 17, 18). Advantages: PAN; 1. Safe to drive after 30 min (13, 20, 43). 2. No anesthesiologist required because all doctors can administer PAN (provided short-hands on training) (7, 8, 17, 36, 37, 40, 41).
Specialized equipment required	Anesthetic machine and anesthetic monitoring equipment (1, 4, 5, 15, 44). Disadvantages: Fixed concentration; 1. Anesthesiologist; 2. Expensive monitoring equipment needed (1, 4, 5, 15, 44).	Less expensive dental equipment only (7, 8, 17, 37). Advantages: PAN; 1. Clinical supervision only and 2. No additional monitoring equipment needed (7, 8, 37).
Duration of exposure	30 (2, 3) and 60 min (1, 4, 5). Disadvantages: Fixed concentration; longer-duration; increases 1. Incidence and 2. Severity of side effects (1–5, 15, 24–26)	Always 20 min (11, 36, 45–48). Advantages: PAN; Shorter duration results in 1. Fewer and 2. Milder side-effects (7, 8, 13, 17).
Patient sensitivity to N_2_O dose	Considered, secondary to goal concentration (1–5, 15). Disadvantages: Fixed concentration; increased incidence of side effects (1, 5, 15); 1. Nausea (7, 8, 26); 2. Vomiting (7, 8, 26)/(aspiration) (13, 43) and 3. Anesthesia (24–26).	Paramount; always titrated using patient sensitivity as guide (7–10, 17, 26, 37, 40). Advantages: PAN; Limits incidence of 1. Nausea, 2. Vomiting and 3. No danger of A. Anesthesia or B. Aspiration (mouth free) (7, 8, 13, 17, 19, 43).
Technique sensitivity and training	Secondary importance–provided anesthetist present (1, 4, 5, 15). Disadvantages: Fixed concentration; Anesthesiologist always required for safety plus another practitioner for psychiatric aspects (1, 4, 5, 15, 18).	Brief hands-on training needed–No anesthesiologist needed (7, 8, 17, 36, 37, 40). Advantages: PAN; any physician or dentist can administer it single-handed provided hands-on training done (7, 8, 17, 36, 37, 40, 41).
Costs and practicality for any medical practice	Expensive–not suitable for average medical or psychiatric practice. Disadvantages: Fixed concentration; Equipment relatively expensive (36) because 1. Anesthesiologist and 2. Monitoring equipment (7, 8, 17) probably best in hospital.	Cheaper and applicable to any medical/dental practice (6, 7, 11, 17, 40). Advantages: PAN; 1. Relatively inexpensive equipment (36, 40); and 2. Only clinical monitoring required from single-handed practitioner (7, 8, 17).

*Numbers as superscripts refer to references in the text*.

*Titrated N_2_O (PAN) concentrations expressed as pharyngeal concentrations (20, 21), while goal concentrations are expressed as such, but clearly produce higher pharyngeal concentrations than for PAN, because of the relative closed or open circuits respectively used during administration*.

*^†^Effect of 1. Semi-open circuit (nasal mask): N_2_O concentrations in the pharynx, substantially lower than rotameter settings (20, 21)*.

2. Semi-closed circuit (full-face mask, strapped to face): N_2_O concentrations in the pharynx, closer to rotameter settings (43).

*Fixed concentrations takes little cognisance of each patient's sensitivity to N_2_O and therefore most patients will be overdosed, with fixed concentrations of N_2_O (7, 8, 17, 24–26)*.

*^††^Despite the statement “Patient recovery is very quick and most patients have fully recovered within 10–15 min” (42), is a good illustration of the lack of practical expertise of those anesthesiologists who do not use procedural analgesia and sedation or PAN regularly (12, 27, 43)*.

## Psychotropic analgesic nitrous oxide (titrated variable concentrations) in psychiatry

PAN has been used as an investigative, diagnostic and therapeutic tool in psychiatry (11) for:-

1. Pain perception. Here, research uncovered an endogenous algesic opioid system that counterbalanced the well-known analgesic system (11, 31–33). An algesic opioid system in dog pontine-medullary area was located by others (33).
2. Human sexual research. It gave tentative evidence of opioid system involvement in human sexual response (49), which was later confirmed more rigorously by others (50). Our work indicated that N_2_O might be useful for treating and researching female sexual dysfuction (49). Further, the action of these two opposing opioid systems uncovered a possible *physiological* link on the pain-pleasure continuum. The existence of such a continuum was first postulated by Aristotle (51) and later espoused by Descartes and Spinoza (52). The pain pleasure system also seemed involved in substance abuse (53–56), as well as the placebo response (34, 45, 57, 58). Moreover, the opioid system could be on a common pathway underlying all substances of abuse (59). Interestingly, later researchers have suggested the opioids, the placebo response, drug addiction and learning are all linked as part of reward and punishment continuum (60).
3. Depression research, both with (10) and without substance abuse (9). We have also showed that the gas could be used to treat depression during the latent period, before conventional antidepressants become effective (9, 61). Further, we have showed the role of the placebo response in the action of antidepressants (62). It is pleasing that Nagele et al. (1, 4, 15) have confirmed our observations that N_2_O is an antidepressant.
4. Investigating various psychiatric conditions. These include inpatient therapy for alcohol abuse (10, 63) as well as the likely possibility that the gas has potential for outpatient alcohol withdrawal treatment (64). Moreover, we have also demonstrated that N_2_O can ameliorate withdrawal from opioids (65), cocaine (46), cannabis (47, 63), nicotine (63), and methaqualone (47). We have also used N_2_O for other conditions such as anxiety (11) stress (48) psychosis (66), eating (67, 68) and movement disorders. The movement disorders studied, include neuroleptic-induced akathisia (69), Tourette Syndrome (70) spasmodic torticollis (71) and hyperactivity (72).
5. Finding a double-blind method of applying rapidly acting agents like N_2_O for future research (35, 73).
6. For discovering in 1983, that N_2_O (31, 32, 38, 39, 74, 75), was the first gas identified as a gaseous neurotransmitter, whereas nitric oxide (NO) was only shown to be a gaseous neurotransmitter in 1990, i.e., 7 years later (76).

## Fixed concentrations of subanesthetic N_2_O in psychiatry

Recently, a group in the USA, showed single-blind (*N* = 20) (1) that N_2_O ameliorates treatment resistant depression and that in a single case (4) that it might have more lasting effects. In 2016, British investigators found evidence that 50% N_2_O might be useful in suppressing traumatic thoughts (2). The latter also observed that the subjective response to 50% N_2_O might be a marker for future alcoholism (3). Later, the Americans published a three-patient case study showing the gas might decrease intrusive traumatic thoughts in post-traumatic stress disorder (5). In 2021 the same USA group (15), demonstrated that 50% N_2_O in oxygen ameliorated depression. They listed 15 unwanted side-effects, among others; dizziness, uncontrolled laughter, feeling of disconnection, paranoia as well as nausea and vomiting. In an effort to reduce the side-effects they tried 25% and found less than half the side effects. When side-effects occurred, these were considerably reduced as compared to the higher concentration (15). For instance, in a single subject only nausea occurred *without* vomiting (15). However, as will be seen later, some sensitive subjects may also vomit at fixed concentrations of 22.5% or less (7).

For these reasons fixed 50% concentrations may be hazardous in general medical or psychiatric practice, unless practitioners are trained anaesthesiologists (1, 5, 6). Indeed, even a 25% concentration may also be undesirable among those untrained as specialist anesthesthesiologists. It is therefore significant that the dental titration technique has been safely used since the 1940's, in routine *general* dental practice (7–9, 11, 17) and since the 1980's in psychiatry (8–12, 34, 45, 57). Concentrations used in dentistry are almost *always* lower (7–11, 17) than those recommended in the most recent research (1–6, 15, 16).

## Differentiating subanaesthetic N_2_O from PAN

### What is PAN

The term PAN was initially introduced to avoid confusion when speaking to medical professionals, about its use in neuropsychiatry and dentistry (12). Most physicians, apart from obstetricians (22), seldom, if ever, use the low concentrations favored in dentistry. And for labor analgesia, 50% N_2_O is mainly administered briefly, intermittently, and on demand only (22). Dentists use low titrated concentrations of the gas (i.e., PAN), as an anxiolytic, while the patient is conscious and fully co-operative (7, 8, 12, 13). Any fixed concentration of N_2_O is discouraged in modern dentistry (7, 8, 12, 17). Confusion occurs, because other medical professionals including anaesthesiologists, usually know the gas as part of balanced anesthesia only. Further, few anaesthesiologists have regular experience of using N_2_O for minimal sedation in conscious patients, apart from those who regularly use procedural sedation and analgesia (44). And then, N_2_O is usually part of a cocktail of other agents.

### Subtle but important differences between PAN and fixed concentrations

The differences are subtle. But are of paramount importance for ethical and practical reasons. Thus, readers must clearly distinguish subanaesthetic N_2_O and PAN, although both fall within Stage 1 Anesthesia. PAN lies within the first half of Stage 1, while a 50% concentration lies in the second half of Stage 1 (19). Indeed, in some N_2_O-sensitive individuals 25% may also lie within the second half of Stage 1 (19). The difference is more relevant now, since the latest work, features the administration of relatively high concentrations of subanaesthetic N_2_O (1–6, 13, 15, 16, 19), *not* consistent with PAN (7, 8, 17, 33).

Unlike PAN these relatively high concentrations are administered *via* a full face-mask i.e., a relatively closed system for 30 (2, 3) or 60 min (1, 4, 5, 15). [I]In addition, the goal concentration seems more important than patient comfort (1–6, 15). In contrast, and by definition, PAN implies titrating N_2_O to the *lowest* levels through a relatively open system (nasal mask) to each subjects clinical *comfort*, while *fully* conscious (7, 8, 10–12, 17, 47). There is definitely no goal concentration. Rather, the goal is maximum patient comfort and anxiolysis (7, 8, 12). For psychiatry, excellent results are obtained after 20 min only (9–11, 47). As a result, there are fewer side-effects, which when they occur, are milder (7–11, 13, 17).

### N_2_O administration *via* open circuit avoids anesthesia and unpleasant side-effects

A crucial result of these difference is that while using a similar semi-closed circuit (1–5), ~30% of subjects become unconscious while breathing 35–45% N_2_O (24–26). Indeed, unconsciousness results even if the gas is applied for less than an hour (13, 24, 25). Further, in those not anesthetised while on a semi-closed facial mask system; and inhaling for considerably shorter periods than 60 min (2, 3), individuals suffer unpleasant side-effects like nausea, vomiting etc. while inhaling <50% (1, 5, 13, 24, 25), or 25% (7, 8). In contrast, correctly applied PAN does not result in anesthesia (7, 8, 13, 17, 19), while other side-effects are milder and less frequent (7, 8, 13). This because, the optimum dosage is evaluated by the practitioner on a case by case basis.

Importantly, like procedural sedation and analgesia (44) the gas is titrated to a clinical state (*not* a predetermined goal concentration) thus, there is no actual or finite concentration, even in the same subject (25) when PAN is correctly used (7, 8, 12, 13, 17, 44). But, once again these concentrations are lower than 50% (20, 21), and fall within the first half of Stage 1 Anesthesia (19).

### Lower titrated N_2_O concentrations prevent anesthesia

There is another basis for the rarity of anesthesia and other untoward side-effects with PAN. Simply, the concentrations of N_2_O are always lower than a 50% (1, 4–6, 15) or 25% (15) fixed mixture administered through a strapped facial mask, because dental flowmeters use a *semi-open* nasal mask system (7, 8, 13, 14) Further, dental flowmeters ensure that the maximum concentration, as per the rotameters is 70% (7, 8). And typical dental nasal-mask systems (7, 8, 12, 17), produce concentrations of N_2_O reaching the pharynx less than half than those shown on the rotameters (20, 21). With PAN, <1%, of subjects require rotameter setting of 70%, which is the maximum concentration possible with a dental flowmeter. Here, the actual alveolar concentration of N_2_O is <35%, in O_2_. In terms of these lower rotameter to pharyngeal concentrations, Malamed indicates that 91% of subjects given 22.5% N_2_O or less will be adequately sedated (7). Consequently, any fixed concentration of 25% (15) or 50% (1–5, 15) will cause numerous individuals to be over-sedated (7), resulting in avoidable side effects (7, 8).

Obviously, for balanced anesthesia, these variable concentration effects when N_2_O is used are of limited import. Here the goal endpoint is rapid unconsciousness and *not* consciousness. Thus, for anesthesia, virtually all subjective effects can be discounted. Of course, this presupposes that the patient has good intra-operative analgesia and there is an absence of awareness, as well as post-operative recall. Clearly, where the gas is used on conscious people their subjective responses *are* of paramount importance.

### Bell-shaped N_2_O sensitivity curve

N_2_O, like any pharmacological agent used in procedural sedation and analgesia, manifests a normal bell-shaped distribution curve (7, 8) reflecting the *sensitivity* of subjects to the agent (7, 8, 12, 19, 44), when given during consciousness (7, 8, 11, 17). And, the dose-response curve is of central importance when administering rapid onset and offset agents like N_2_O to *conscious* subjects (7, 8, 12, 17, 19, 25, 26). Furthermore, the dose-response curve in any individual, can vary even from day to day, because it is state rather than trait dependant (25, 77).

### Differences in receptor actions of titrated vs. fixed concentration N_2_O

Apart from dose response differences, there is another important distinction. The mechanisms underlying PAN differs from higher subanaesthetic concentrations. For instance, the analgesic properties of subanaesthetic N_2_O and PAN are mediated mainly by the endogenous opioid and GABA-ergic system (10, 11, 29, 31, 32, 38, 39). In contrast, the anesthetic actions of the gas occur mainly through N-methyl-D-aspartate receptor (NMDAR) (29, 30). As we have seen, some patients may be inadvertently anesthetised, when using a fixed 50:50 mixtures of N_2_O (7, 8, 13, 17, 24–26). Thus, the underlying mechanisms governing the consciously experienced psychotropic actions of N_2_O, are different to those where pre-anesthesia or anesthesia supervene. Consequently, one can see how the antidepressant actions of N_2_O could *wrongly* be attributed to the NMDAR blockade (1–5, 15, 27, 78). Indeed, the same error has been made regarding ketamine, which until recently (27, 28, 79), was purported to have been due to NMDARs (1–5, 42).

Although convenient, and *superficially* more scientific to use fixed concentrations of 25% (15) or 50% (1, 2, 4, 5, 15) N_2_O in oxygen, it may be imprudent to generalize findings with these concentrations. Particularly, because of the higher concentrations delivered by a relatively closed system. A factor complicated by the varying sensitivity of patients to N_2_O (7, 8, 17, 24–27). These variables are virtually eliminated where the concentration is correctly titrated to patient comfort levels (PAN) (11, 12, 17, 27) as compared to higher subanaesthetic goal concentrations (1–5, 15). Moreover, the mechanism involved underlying observations (1–5, 15) may also be questionable (27, 78), even where the operator chooses to titrate to a highish goal concentration (1, 5, 24, 27, 28, 78). For these reasons, the use of continuous-flow, fixed concentrations of 25% (15) or 50% N_2_O (1–6, 15), are inappropriate (7, 8, 12, 13, 24–27, 77).

### Side effects prevented by titration

Almost all these unwanted side effects, particularly inadvertent unconsciousness and/or nausea and vomiting are avoided by using the correct dental technique (7, 8, 13, 17). Here, the gases are titrated to each subjects' unique requirements, always using a nasal (7–12, 17), rather than a full-face (1–5, 15) mask. To use the dental technique safely, effectively, and correctly, a *shor*t hands-on training (lasting a few hours), is essential (36, 37, 40, 41).

From the foregoing it should be clear, that those using a 25% or 50% goal gas concentration mixture (1–5, 15) for periods above a few minutes, must therefore accept that unpleasant side-effects, such as inadvertent anesthesia, nausea, vomiting and inappropriate affective changes as *inevitable* in some cases (1, 7, 8, 13, 24–26, 43). A fact borne out in two studies, where nausea and vomiting has already been noted in subjects breathing 50% N_2_O (1, 5, 15). These problems are almost always avoided when N_2_O is titrated to each subjects' requirements (7, 8, 11, 12, 17, 40), so that the subject is always conscious and co-operative. For these reasons, readers will understand the practical importance of distinguishing between subanaesthetic and psychotropic concentrations of N_2_O.

### Confusion of PAN with discontinued use of 100% N_2_O for anesthesia

In the 19th and early 20th Centuries, 100% (hypoxic) N_2_O was used for anesthesia (13, 80) which was potentially fatal, (80) possibly producing some deaths. Although, since the 1st years of the last century it was abandoned in medicine and dentistry, the confusion exists today. After training dentists and physicians in the PAN technique over 40 years, I am regularly told by potential trainees or anesthetists that the technique is dangerous. These people invariably confuse PAN with using 100% N_2_O for anesthesia. It would be a pity, if PAN *wrongly* fell into disrepute, because an untrained practitioner, using 25% or 50% N_2_O goal concentrations plus a full-face mask, produced a fatal pulmonary aspiration (43).

Although deaths are unlikely even when used at 50% or 25% (with a relatively closed circuits), unpleasant affective and physical side-effects are unavoidable in a certain percentage of patients (7, 8, 13, 15, 17, 24–26, 43), including vomiting, nausea and others, as confirmed recently (1, 5, 6, 15).

As discussed in some detail above, these high subanesthetic concentrations can and do, produce unconsciousness, vomiting, nausea and other disagreeable effects (1, 5, 6, 13, 24–26). Until the single controlled double-blind study (15) is repeated by others, these studies show promise only. Nonetheless, they do support the earlier work showing that PAN is antidepressant (9, 10). Interestingly, the same American group have published 3 reviews (23, 42, 81), heavily favoring the NMDAR and practically ignoring the other neurotransmitter systems, notably the opioid system (27).

Sadly, even if more studies are undertaken and confirm the north American work (15), the possibility of using fixed concentrations of 50% N_2_O in general psychiatric or medical practice are limited. Such studies are limited unless an anesthesthesiologist administers the gas and we accept that many subjects will be oversedated. The evidence mentioned above, also clearly indicates that a 25% fixed dose is also unsuitable. Nonetheless, the realization that N_2_O 25% (15) is probably better than 50% (15) seems an advance, particularly if these researchers realize the advantages of the PAN titration technique and to begin to use it. However, at this stage, the authors (1, 4, 6, 15) are still using arbitrary dosage rather than a dose optimized to each specific patient's needs.

## Conclusions

At the moment, there is little controlled evidence showing the efficacy (42) and safety of subanesthetic fixed doses of N_2_O in in mood disorders. Nonetheless, the gas does show promise for mood and other psychiatric disorders (11, 15). Indeed, it is only comparatively recently that an adequate double-blind method has been devised using N_2_O (35, 73). The latter method is able to prevent the identification of the gas by both subjects and investigators (1, 15).

Compelling ethical and practical reasons make it unwise or even dangerous for psychiatrists or any other practitioner, unqualified as an anesthesthesiologists, to use fixed concentrations of 50% N_2_O (with full face mask), by continuous flow. The only way that continuous flow N_2_O at 50% can be safely used is when an anesthesiologist is present. The use of fixed 25% N_2_O should also be avoided, to prevent unnecessary suffering (7, 8, 15).

In closing, I leave the reader with this question: Is the current method, where fixed goal concentration are advocated (1–6, 15), going to result in a useful agent falling into disrepute because of unnecessary patient suffering or other unintended harmful consequence? A particular problem in the hands of those not qualified as anesthesiologists.

## Data availability statement

The original contributions presented in the study are included in the article/supplementary material, further inquiries can be directed to the corresponding author.

## Author contributions

The author confirms being the sole contributor of this work and has approved it for publication.

## Conflict of interest

MG is a medical adviser to Sedatek.

## Publisher's note

All claims expressed in this article are solely those of the authors and do not necessarily represent those of their affiliated organizations, or those of the publisher, the editors and the reviewers. Any product that may be evaluated in this article, or claim that may be made by its manufacturer, is not guaranteed or endorsed by the publisher.
